# Epigenetic age oscillates during the day

**DOI:** 10.1111/acel.14170

**Published:** 2024-04-18

**Authors:** Karolis Koncevičius, Akhil Nair, Aušrinė Šveikauskaitė, Agnė Šeštokaitė, Auksė Kazlauskaitė, Audrius Dulskas, Artūras Petronis

**Affiliations:** ^1^ Institute of Biotechnology, Life Sciences Center, Vilnius University Vilnius Lithuania; ^2^ The Krembil Family Epigenetics Laboratory, The Campbell Family Mental Health Research Institute, Centre for Addiction and Mental Health Toronto Ontario Canada; ^3^ Laboratory for Genetic Diagnostics National Cancer Institute Vilnius Lithuania; ^4^ Department of Abdominal and General Surgery and Oncology National Cancer Institute Vilnius Lithuania; ^5^ Faculty of Medicine Vilnius University Vilnius Lithuania

**Keywords:** circadian rhythms, DNA modification, epigenetic age, epigenetic clocks, WBC subtype heterogeneity

## Abstract

Since their introduction, epigenetic clocks have been extensively used in aging, human disease, and rejuvenation studies. In this article, we report an intriguing pattern: epigenetic age predictions display a 24‐h periodicity. We tested a circadian blood sample collection using 17 epigenetic clocks addressing different aspects of aging. Thirteen clocks exhibited significant oscillations with the youngest and oldest age estimates around midnight and noon, respectively. In addition, daily oscillations were consistent with the changes of epigenetic age across different times of day observed in an independant populational dataset. While these oscillations can in part be attributed to variations in white blood cell type composition, cell count correction methods might not fully resolve the issue. Furthermore, some epigenetic clocks exhibited 24‐h periodicity even in the purified fraction of neutrophils pointing at plausible contributions of intracellular epigenomic oscillations. Evidence for circadian variation in epigenetic clocks emphasizes the importance of the time‐of‐day for obtaining accurate estimates of epigenetic age.

## Graphical abstract

In this article, we report an intriguing finding: epigenetic age predictions display a 24‐h periodicity. Numerous epigenetic clocks consistently showed that human blood cells appeared much younger around midnight compared to noon. This finding emphasizes the importance of considering the time of‐day when estimating epigenetic age.

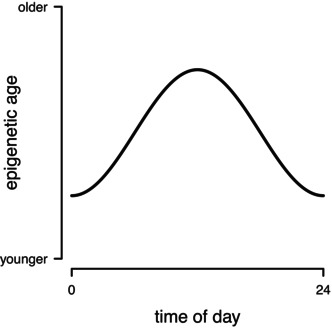

**Abbreviations**
MESOR: Midline estimating statistic of rhythm
modC: modified cytosines
NK‐cells: natural killer cells
PBMC: peripheral blood mononuclear cells
PC: principal component
WBC: white blood cell

Epigenetic clocks use cytosine modification (methylation) densities to estimate chronological and biological age (Bernabeu et al., 2023). These clocks have quickly gained popularity and found applications in disease studies (Dugué et al., 2018; Grant et al., 2017; Roetker et al., 2018), prediction of all‐cause mortality (Zhang et al., 2017), forensic medicine (Guan et al., 2021), and are even marketed commercially for monitoring epigenetic response to lifestyle alterations (Dupras et al., 2020). In addition, epigenetic clocks are also used to estimate cumulative stem cell divisions for cancer risk prediction (Teschendorff, 2020; Yang et al., 2016) and telomere length (Lu, Seeboth, et al., 2019).

The majority of the aging studies investigating epigenetic clocks use whole blood as the tissue of interest. However, experiments in our lab (Oh et al., 2019) and from other groups (Aroca‐Crevillén et al., 2020; Born et al., 1997) have shown that white blood cell (WBC) subtype counts and their proportions oscillate with a 24 h periodicity. The cycling counts of neutrophils, lymphocytes, and other WBC subtypes can create evidence for oscillations of modified cytosines (modC) in whole blood, even when epigenomes of individual subtypes remain static. In turn, these pseudo‐oscillating cytosine modifications may induce cyclical variations in epigenetic age. In addition to this intercellular periodicity of WBC composition, evidence for 24 h oscillations of modCs was also detected intracellularly in purified human WBCs (Oh et al., 2019). Cytosines which exhibited daily intracellular epigenetic oscillations tended to overlap with cytosines that linearly changed their modification density with age (ibid.), hinting at their putative contributions to the epigenetic age estimates.

To test the hypothesis that epigenetic clocks are influenced by either inter or intracellular modC rhythmicity, or both, we performed a comprehensive analysis of several circadian WBC samples (Figure S1). First, we re‐analyzed our dataset which consisted of blood samples collected every 3 h for a period of 72 h from a healthy 52‐year‐old male (Oh et al., 2019). The WBCs were divided into two parts: neutrophils purified using magnetic activated cell sorting and the remaining WBCs depleted of neutrophils (WBC‐Neu). WBC‐Neu samples still contained a non‐negligible proportion of neutrophils (~10%–20% of all WBCs) (Table S1, Figure S2) and resemble peripheral blood mononuclear cells (PBMC) which are frequently used in human epigenomic studies. Having daily epigenetic variation measured in WBC‐Neu and neutrophils from the same individual offers us a unique perspective on epigenetic age dynamics in the “whole vs. part.” Cytosine modifications were profiled using Illumina HumanMethylation arrays (see methods).

In the WBC‐Neu fraction, we identified 58,489 modC sites oscillating with a period of 24 h (cosinor *p* < 0.05), 62 of which overlapped with the 353 (17.5%) cytosines comprising the Horvath pan‐tissue 2013 clock. Similarly, oscillating modCs were also detected in other age predictors, for example, 23 of 71 (32.4%) and 24 of 99 (24.2%) modCs exhibited oscillations in the Hannum 2013 and Lin 2016 clocks, respectively.

We then obtained epigenetic age estimates using an online DNAmAge calculator (Horvath, 2013) and R package “methylCIPHER” (Thrush et al., 2022). Five out of seven chronological age clocks showed significant age oscillations over the 24 h period in the WBC‐Neu dataset (Figure 1a,d). In addition, all four mitotic‐like clocks (Figure 1b,d), and four out of six biological age clocks (Figure 1c,d) also showed statistically significant 24 h oscillations. In total 13 out of 17 clocks tested showed significant oscillations, eight of which remained significant after Bonferroni correction for multiple testing (Figure 1d).

**FIGURE 1:**
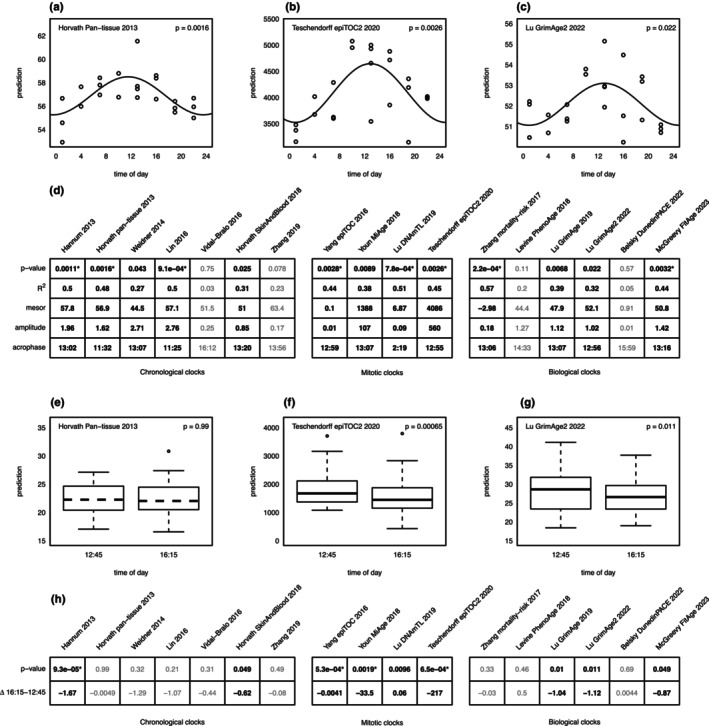
Epigenetic age oscillations in blood. (a–c) 24 h dynamics of epigenetic age predictions in WBC‐Neu dataset for Horvath pan‐tissue 2013 (a), Teschendorff epiTOC2 2020 (b), and Lu GrimAge2 2022 (c) clocks. Lines depict cosinor regression fits with solid lines indicating oscillation significance (cosinor *p* < 0.05). x‐axis: time of day; y‐axis: epigenetic age. (d) Estimated oscillation parameters of the 17 tested epigenetic clocks. Bold values indicate significant oscillations (*p* < 0.05), and asterisks indicate significance after Bonferroni correction for multiple testing. (e–g) Boxplots of epigenetic age predictions at two time points: 12:45 and 16:15 (Apsley et al., 2023) for Horvath pan‐tissue 2013 (e), Teschendorff epiTOC2 2020 (f), and Lu GrimAge2 2022 (g) clocks. Solid median lines indicate statistically significant differences (*p* < 0.05, paired Student's *t*‐test). x‐axis: time of day; y‐axis: epigenetic age. (h) Paired Student's *t*‐test *p*‐values and pairwise mean differences of epigenetic age between 12:45 and 16:15, matched by donor.

All oscillating clocks followed a similar pattern—their age and mortality predictions reached the maximum (acrophase) and minimum (nadir) around noon and midnight, respectively. For example, according to Horvath pan‐tissue 2013 clock, the average predicted epigenetic age of the 52 year old individual oscillated from 55.3 years at ~11:30 PM to 58.5 years at ~11:30 AM (cosinor *p* = 1.6 × 10^−3^; Figure 1a). Lu DNAmTL 2019 clock telomere estimates were “longest” after midnight (~2AM) which, in line with all other clocks, indicates the “youngest” epigenetic age. The peak to trough age‐range for chronological clocks varied from 1.7 yrs in Horvath SkinAndBlood 2018 clock to 5.5 years in the Lin 2016 clock. Two out of four non‐oscillating clocks (Levine PhenoAge 2018 and Zhang 2019) demonstrated similar acrophases to the oscillating clocks with relatively low *p*‐values (0.11 and 0.078, respectively; Figure 1d) suggesting a weaker effect and a lack of statistical power.

We also tested recently developed principal component (PC) versions of epigenetic clocks, which integrate epigenetic aging signals shared across numerous cytosines (Higgins‐Chen et al., 2022). Five out of six PC clocks exhibited significant oscillations (Figure S3). All oscillating PC clocks followed similar trends as the non‐PC clocks, showing the oldest and youngest age estimates at noon and midnight, respectively. For example, according to the PC version of the Horvath pan‐tissue clock, the predicted age of the 52 year old individual oscillated from 52.4 years at ~12:15 AM to 54.8 at ~12:15 PM. Furthermore, the oscillations in the PC clocks were even more pronounced compared with their non‐PC counterparts, likely due to the former being more robust against technical noise (Figure S3).

In populational epigenomic studies where samples are usually collected during regular work hours, vestiges of epigenetic age oscillations should be detectable as time‐of‐day effects. To test this, we re‐analyzed modC profiles of PBMC collected from the same individuals at four separate time points spanning a ~5 h period (Apsley et al., 2023). We estimated pairwise epigenetic age differences between 12:45 PM (the second measurement in the study, which was the closest available time point to the observed acrophase of epigenetic age in WBC‐Neu collection) and the latest available time point at 4:15 PM in 32 matched sample pairs. Consistently with the expected decrease of oscillating epigenetic age towards the evening, 15 of 17 clocks returned lower estimates at 4:15 PM compared to 12:45 PM, with nine clocks reaching statistical significance (Figure 1h). In addition, all six PC‐based versions of the clocks followed similar patterns and returned statistically significant younger ages at 4:15 PM (Figure S4). The original study (Apsley et al., 2023) investigated the epigenetic effects of acute stress which were not taken into account in our re‐analysis. However, it is highly unlikely for stress to induce a rapid “rejuvenation”, and therefore the observed differences are more likely to reflect genuine time‐of‐day effects.

What are the sources of epigenetic age oscillations? Initial clues came from the WBC composition analysis. We estimated the subtype makeup of WBC‐Neu samples using the Houseman's method (Houseman et al., 2012) and detected that the proportions of natural killer cells (NK‐cells), B and CD4+ T lymphocytes oscillated with a 24 h periodicity (Figure 2a,b). Around midnight, corresponding to the epigenetic age nadir, the proportion of NK‐cells decreased to their minimum, while B and CD4+ T lymphocytes reached their maximums (Figure 2a,b). Next, we asked if these oscillating WBC subtypes could have contributed to the observed periodicity of epigenetic age. To this end, we re‐analyzed two datasets (Reinius et al., 2012; Wang et al., 2023) containing samples of whole blood as well as six sorted WBC subtypes and estimated their epigenetic ages using Horvath pan‐tissue 2013 clock. Compared to the whole blood, the clock predicted older ages for NK‐cells (mean +/−sd = +3.59 +/−5.15) but returned younger ages for B‐ and CD4+ T lymphocytes (mean +/‐sd = −4.46 +/− 4.44 and −3.99 +/− 4.12 years, respectively) (Figure 2c,d). Could these three WBC subtypes influence the epigenetic age predictions in whole blood? We re‐analyzed a populational epigenetic aging dataset (Johansson et al., 2013) and detected that the proportion of NK‐cells indeed correlated with older epigenetic age (Pearson's *r* = 0.14, *p* = 0.0034), while higher proportions of B and CD4+ T lymphocytes were associated with younger epigenetic age (Pearson's *r* = −0.17 and −0.19; *p* = 5 × 10^−4^ and 6.3 ×10^−5^, respectively) (Figure 2e–g). Similar results were obtained for other epigenetic clocks. However, WBC subtype biases varied in size and direction (age accelerating or decelerating), depending on the unique collections of cytosines utilized by each clock (Figures S5–S7).

**FIGURE 2:**
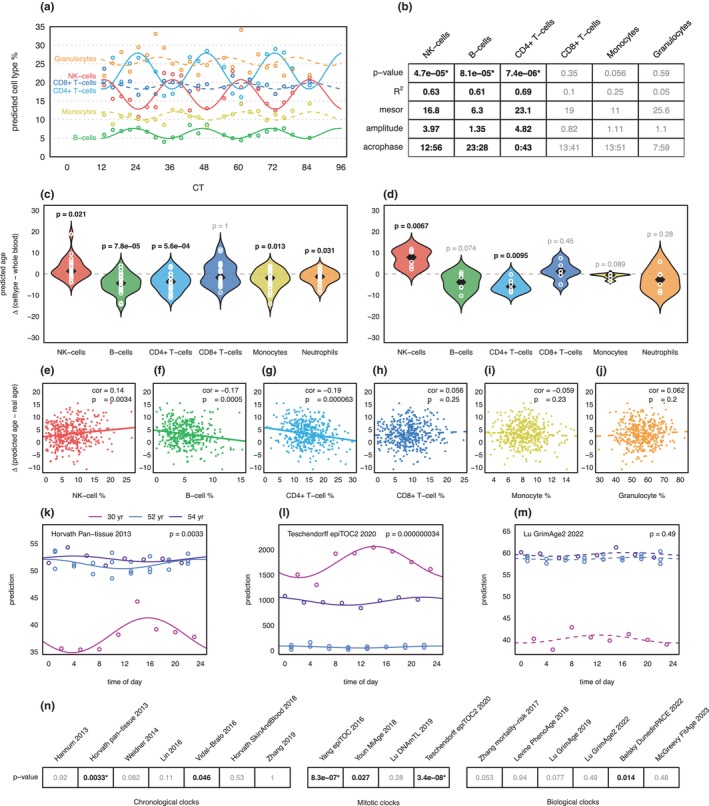
Epigenetic age in WBC subtypes. (a) Oscillations of estimated WBC subtype proportions in WBC‐Neu dataset. Lines depict cosinor regression fits. Solid lines indicate oscillation significance (cosinor *p* < 0.05). x‐axis: blood sample collection time; y‐axis: predicted proportion of WBC subtypes. (b) Estimated oscillation parameters of the six WBC subtypes. Bold values indicate significant oscillations (*p* < 0.05), and asterisks indicate significance after Bonferroni correction for multiple testing. (c, d) Violin plots of epigenetic age differences according to Horvath pan‐tissue 2013 clock between WBC subtypes and matched whole blood samples in Wang et al. (Wang et al., 2023) (c) and Reinius et al. (Reinius et al., 2012) (d) datasets. Numbers above violin plots depict *p*‐values (bold—*p* < 0.05) of Student's paired *t*‐test between each WBC subtype compared to whole blood, matched by donor. x‐axis: WBC subtype; y‐axis: epigenetic age deviation of WBC subtype from whole blood. (e–j), Correlations between epigenetic age deviation and estimated WBC subtype proportion in the whole blood (Johansson et al., 2013). Lines depict linear regression slopes. Solid lines indicate significant Pearson's correlations (*p* < 0.05). x‐axis: predicted WBC subtype percentage; y‐axis: epigenetic age deviation from real chronological age. (k–m) Epigenetic age predictions in neutrophil datasets using Horvath pan‐tissue 2013 (k), Teschendorff epiTOC2 2020 (l), and Lu GrimAge2 2022 (m) clocks. Lines depict cosinor regression fits; colors represent different individuals. Solid lines indicate significant combined analysis oscillations (*p* < 0.05). x‐axis: time of day; y‐axis: epigenetic age. (n) Cosinor *p*‐values of 17 tested epigenetic clocks. Bold values indicate significant oscillations (*p* < 0.05), and asterisks indicate significance after Bonferroni correction for multiple testing.

Differential contributions of WBC subtypes to epigenetic age is not a new observation, and earlier studies exploring blood cell composition (primarily T‐lymphocytes) separated the epigenetic aging into extrinsic and intrinsic components (Horvath et al., 2016; Horvath & Raj, 2018). The former is supposed to capture the aspects of immune‐senescence and disease‐dependent variability in WBC composition. On the contrary, measures of intrinsic epigenetic age aim to take WBC subtype composition into account in order to capture the age‐associated processes inherent within a cell.

The cell count adjustment strategy of regressing epigenetic age on chronological age and a selected subset of predicted WBC counts (Chen et al., 2016) (see methods) reduced epigenetic age oscillations in the WBC‐Neu dataset to non‐significant levels (Figure S8). However, the 12:45–16:15 comparison of PBMC in the populational dataset (Apsley et al., 2023) still exhibited significant time‐of‐day differences in four clocks (Figure S9). These findings suggest that WBC count correction may not be sufficient and/or that epigenetic age oscillations are not exclusively driven by cellular proportions.

The latter interpretation gained experimental support from findings in a purified WBC subtype. We tested three purified neutrophil sets: neutrophils from the donor of WBC‐Neu samples and two additional sets from healthy males aged 30 and 54 years collected every 3 h for a 24 h period (Figure S10,S11). Since circadian parameters showed interindividual differences, we performed a combined analysis allowing for individual‐specific MESOR (Midline estimating statistic of rhythm), amplitude, and acrophase estimates (see methods). Six epigenetic clocks displayed significant 24 h oscillations (cosinor *p* < 0.05) of their predictions, and three of them remained significant after Bonferroni correction for multiple testing (Figure 2n). The most significant evidence for 24 h oscillations was detected for mitotic clocks (Figure 2n). In the 30 year old individual the amplitude of Teschendorff epiTOC2 2020 clock reached 17.6% of the MESOR (309/1756; Figure 2l, Figure S12) indicating that mitotic age estimates can vary by more than 35% (peak to nadir) during the 24 h period.

In summary, our findings indicate that age predictions of epigenetic clocks oscillate throughout the day. Evidently, accounting for daily variation of WBC subtypes in epigenetic aging studies may become obligatory. However, there is a lack of consensus about which chronological and biological age predictors require corrections or which specific WBC subtypes need to be taken into account. Moreover, corrections for WBC counts come at the risk of reducing the clocks' informativeness, as extrinsic measures of epigenetic age were found to exhibit stronger associations with disease and mortality risk compared to their intrinsic counterparts (Horvath et al., 2016).

WBC correction might not be able to fully account for all epigenetic clock oscillation effects. As demonstrated in our study, adjustment for predicted WBC counts resulted in reducing oscillations to non‐significant levels in the WBC‐Neu dataset but the same strategy did not eliminate all time‐of‐day effects in a populational study. Furthermore, we were still able to detect epigenetic age oscillations in purified neutrophils, which is devoid of WBC subtype induced variability. Finally, WBC count adjustment requires adding estimated cell type proportions as covariates to a linear regression model and therefore is restricted to relatively large studies. Clinical, forensic, or personal‐use applications, where the number of samples is less than the number of adjustable WBC subtypes, cannot be subjected to WBC count correction and therefore will be confounded by the time‐of‐day effects.

Populational studies that do not take sample collection times into account would exhibit an increased variability of their epigenetic age estimates which subsequently might reduce their statistical power. Moreover, a mismatch in sample collection times could distort the magnitude of investigated biological effects or even generate false positives. The observed magnitude of epigenetic age oscillations are comparable to the putative age deviations detected in clinical studies. For instance, in a meta‐analysis of epigenetic age acceleration, schizophrenia was reported to demonstrate an average epigenetic age decrease of 0.47 years, according to the Horvath pan‐tissue 2013 clock (Oblak et al., 2021). This estimate is ~7 times lower compared to our observed oscillation range of 3.24 years in the same clock. Similarly, lifestyle interventions were found to reduce Lu GrimAge 2019 predictions by 0.66 and 0.86 years (Moqri et al., 2023), which is less than a half of its daily ~2 year oscillation range.

In this study, epigenetic clocks that estimate the mitotic age (epiTOC, epiTOC2, and MiAge) demonstrated the most consistent oscillations across all datasets. These clocks are based on cytosines located in the promoters of the polycomb repressive complex‐2 genes which play a critical role in ontogeny, cell differentiation, and carcinogenesis (Teschendorff, 2020; Yang et al., 2016). In addition, polycomb‐binding sites are enriched with age‐correlated modCs across multiple tissues and species, and have been recently used to develop a pan‐mammalian epigenetic clock (Lu et al., 2023). Epigenetic oscillations within regulatory elements of the polycomb genes and binding sites point to a new connecting link between development, aging, circadian rhythms, cell cycle, cancer, and chronoepigenetics.

This first effort to examine the 24 h dynamics of the epigenetic clocks has some evident limitations. Investigation of WBCs depleted of neutrophils, the largest WBC subtype whose proportion is antiphasic to lymphocytes (Oh et al., 2019), may have diminished their cellular contribution to epigenetic age predictions. The use of a single individual allowed us to track the fluctuations of estimated epigenetic age without the impact of other biological factors, however, this prohibited us from making generalizations about interindividual differences. The neutrophil findings may be confounded by plausible neutrophil heterogeneity which—similarly to major WBC subtypes—may also exhibit 24 h oscillations. Despite these limitations, our results indicate that failure to account for daily oscillations may hamper estimates of epigenetic age compromising reproducibility and interpretation of the results.

## AUTHOR CONTRIBUTIONS

KK, AN, and AP conceptualized the idea. AN designed the experimental protocol and performed experiments with sample collections and processing assistance from ASe and AK under supervision of AD. KK and ASv performed data analysis. KK, AN, and AP wrote the manuscript. All authors have reviewed and approved the final version of the manuscript.

## FUNDING INFORMATION

This work was supported by the Lithuanian Science Foundation National research program “Healthy ageing” (P‐SEN‐20‐19) and, in part, by the Krembil Foundation, Toronto, Canada, the Future Biomedicine Charity Fund, Vilnius, Lithuania, and by Research Council of Lithuania under the Programme “University Excellence Initiatives” of the Ministry of Education, Science and Sports of the Republic of Lithuania (No. 12‐001‐01‐01‐01 “Improving the Research and Study Environment”). Project No S‐A‐UEI‐23‐10. AP is a Marius Jakulis Jason Foundation scholar.

## CONFLICT OF INTEREST STATEMENT

The authors have no conflict of interest to declare.

## Supporting information


Figures S1–S12.

Table S1.

Data S1.

## Data Availability

Raw Illumina HumanMethylation array files generated by this study are available via the Gene Expression Omnibus (GEO) under accession numbers GSE247197 (WBC‐Neu, 52 yr old), GSE247195 (neutrophils, 54 yr old), and GSE247193 (neutrophils, 30 yr old). Neutrophil dataset for the 52 yr old participant was published previously (Oh et al., 2019) and can be obtained through GEO via accession number GSE83944. The raw data files for all the re‐analyzed publicly available datasets are also accessible through GEO: GSE227809 (PBMC at 4 time points, Apsley et al. (Apsley et al., 2023)), GSE35069 (WBC subtypes, Reinius et al. (Reinius et al., 2012)), GSE224807 (WBC subtypes, Wang et al. (Wang et al., 2023)), and GSE87571 (Whole blood, Johansson et al. (Johansson et al., 2013)).
