# Supplementary material for: Epigenetic age oscillates during the day
Source: Aging Cell. 2024 Apr 18;23(7):e14170. doi: 10.1111/acel.14170 (PMC11258449; doi:10.1111/acel.14170)
Supplement: Supplementary file 1 — Figures S1–S12. [file ACEL-23-e14170-s003.pdf]

Supplement Figures

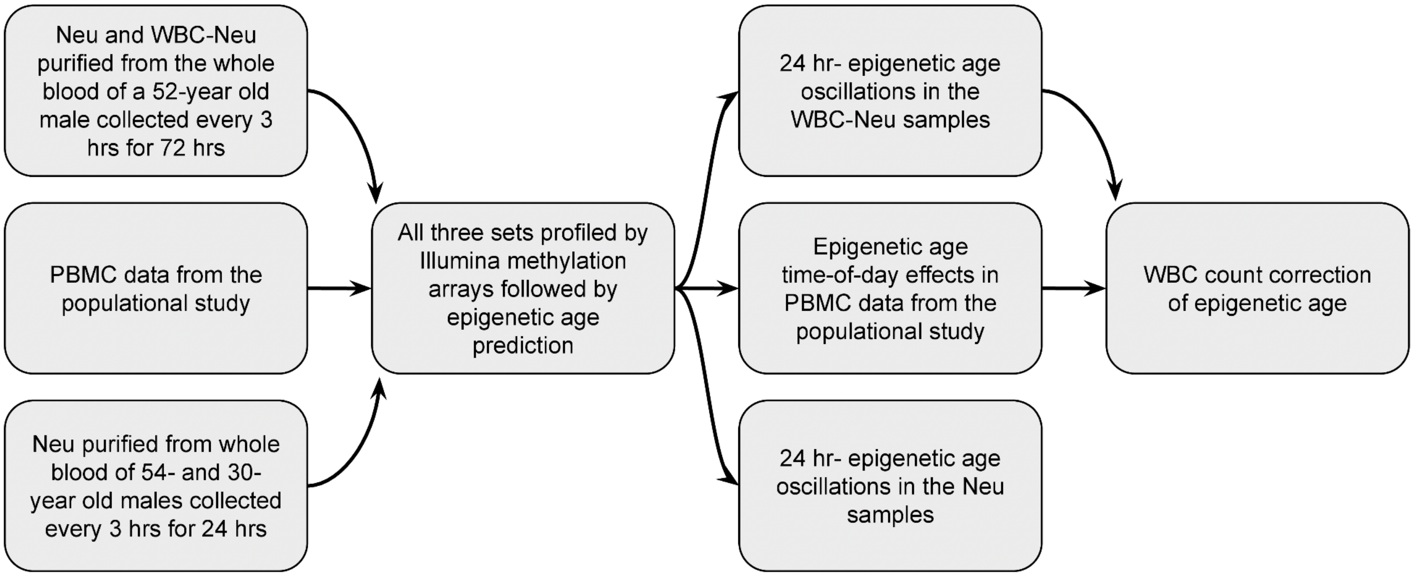

Figure S1 Workflow of the study showing diurnal oscillations of epigenetic age. WBC - white blood cells, Neu - neutrophils, PBMC - peripheral blood mononuclear cells.

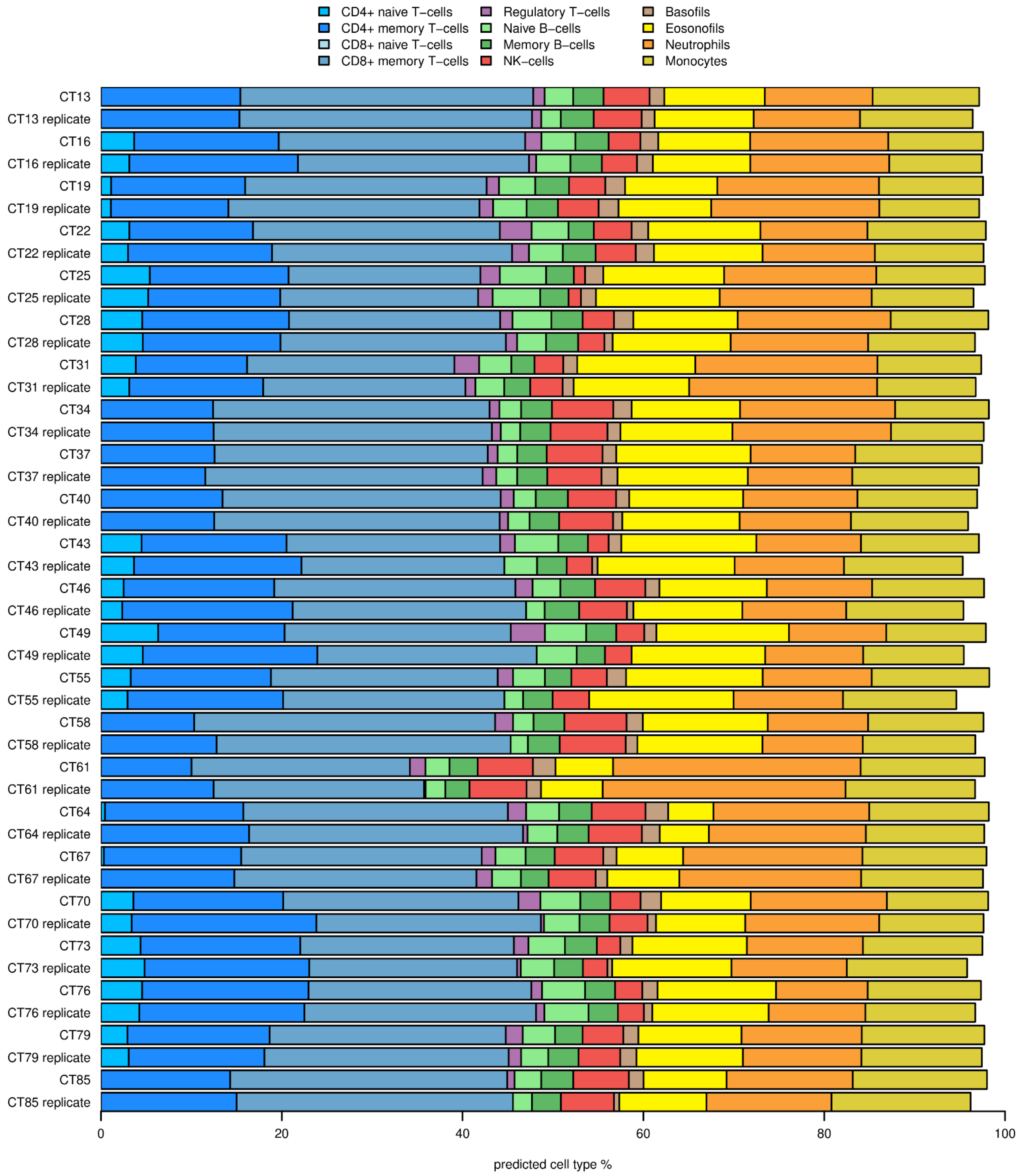

Figure S2 Cell type composition of WBC-Neu samples. Barplot of WBC subtype proportions estimated using Houseman's method (Houseman et al. 2012) with "enhanced cell deconvolution" referenced by Salas et al. (Salas et al. 2022). Colors represent different WBC subtypes. x-axis: estimated cell type proportion in percentages; y-axis: samples arranged by collection time.

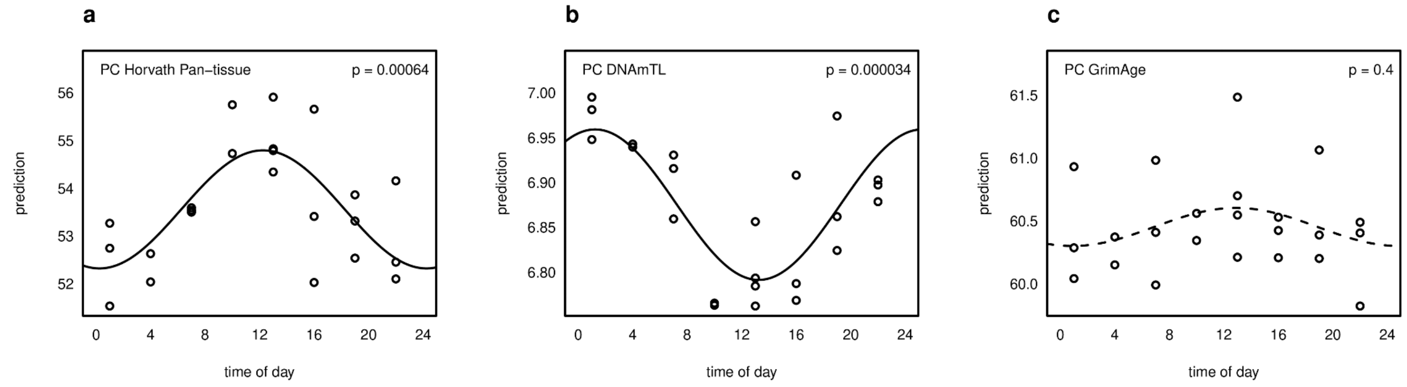

d

	PC Horvath pan–tissue	PC Horvath SkinAndBlood	PC Hannum	PC PhenoAge	PC DNAmTL	PC GrimAge
p–value	**6.4e–04***	**6.7e–05***	**1.8e–04***	**5.2e–04***	**3.4e–05***	0.4
R^2	**0.52**	**0.62**	**0.58**	**0.53**	**0.64**	0.09
mesor	**53.6**	**55.4**	**57.6**	**45.9**	**6.88**	60.5
amplitude	**1.24**	**1.95**	**1.88**	**1.45**	**0.08**	0.15
acrophase	**12:15**	**12:33**	**12:10**	**12:19**	**1:15**	12:53

PC–based clocks

Figure S3 Epigenetic age oscillations for PC-based clocks. (a-c) 24 hr dynamics of epigenetic age predictions in WBC-Neu dataset for PC Horvath pan-tissue (a), PC DNAmTL (b), and PC GrimAge (c) clocks. Lines depict cosinor regression fits with solid lines indicating oscillation significance (cosinor $p < 0.05$). x-axis: time of day; y-axis: epigenetic age. (d) Estimated oscillation parameters of the 6 tested epigenetic clocks. Bold values indicate significant oscillations ($p < 0.05$), and asterisks indicate significance after Bonferroni correction for multiple testing.

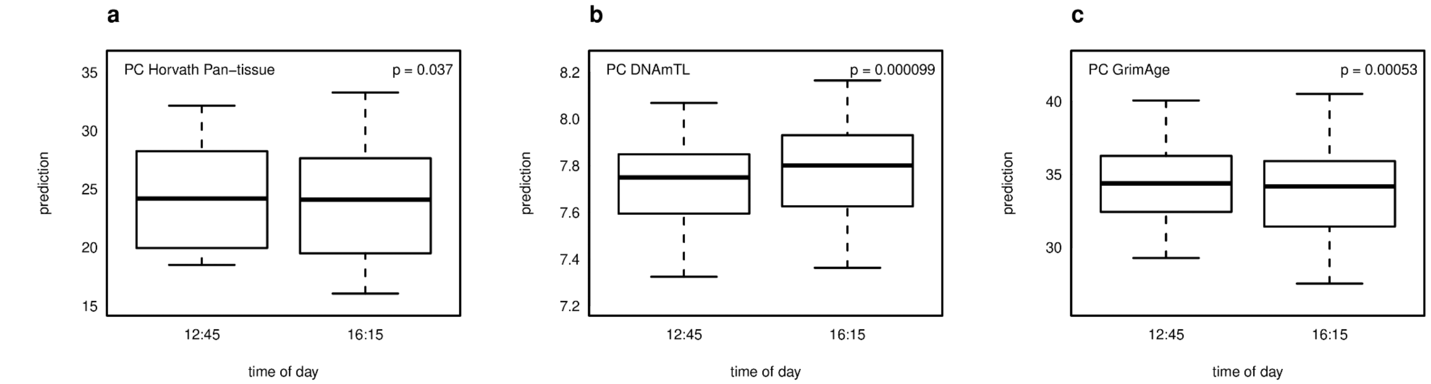

d

	PC Horvath pan−tissue	PC Horvath SkinAndBlood	PC Hannum	PC PhenoAge	PC DNAmTL	PC GrimAge
p−value	**0.037***	**0.0064***	**2.6e−04***	**0.0023***	**9.9e−05***	**5.3e−04***
Δ 16:15−12:45	**−0.54**	**−0.67**	**−1.5**	**−1.28**	**0.05**	**−0.68**

PC−based clocks

Figure S4 Time of day effects for PC-based clocks. Boxplots of epigenetic age predictions at two time points: 12:45 and 16:15 (Apsley et al. 2023) for PC Horvath pan-tissue (e), PC DNAmTL (f), and PC GrimAge (g) clocks. Solid median lines indicate statistically significant differences ($p < 0.05$, paired Student's t-test). x-axis: time of day; y-axis: epigenetic age. (h) Paired Student's t-test p-values and pairwise mean differences of epigenetic age between 12:45 and 16:15, matched by donor.

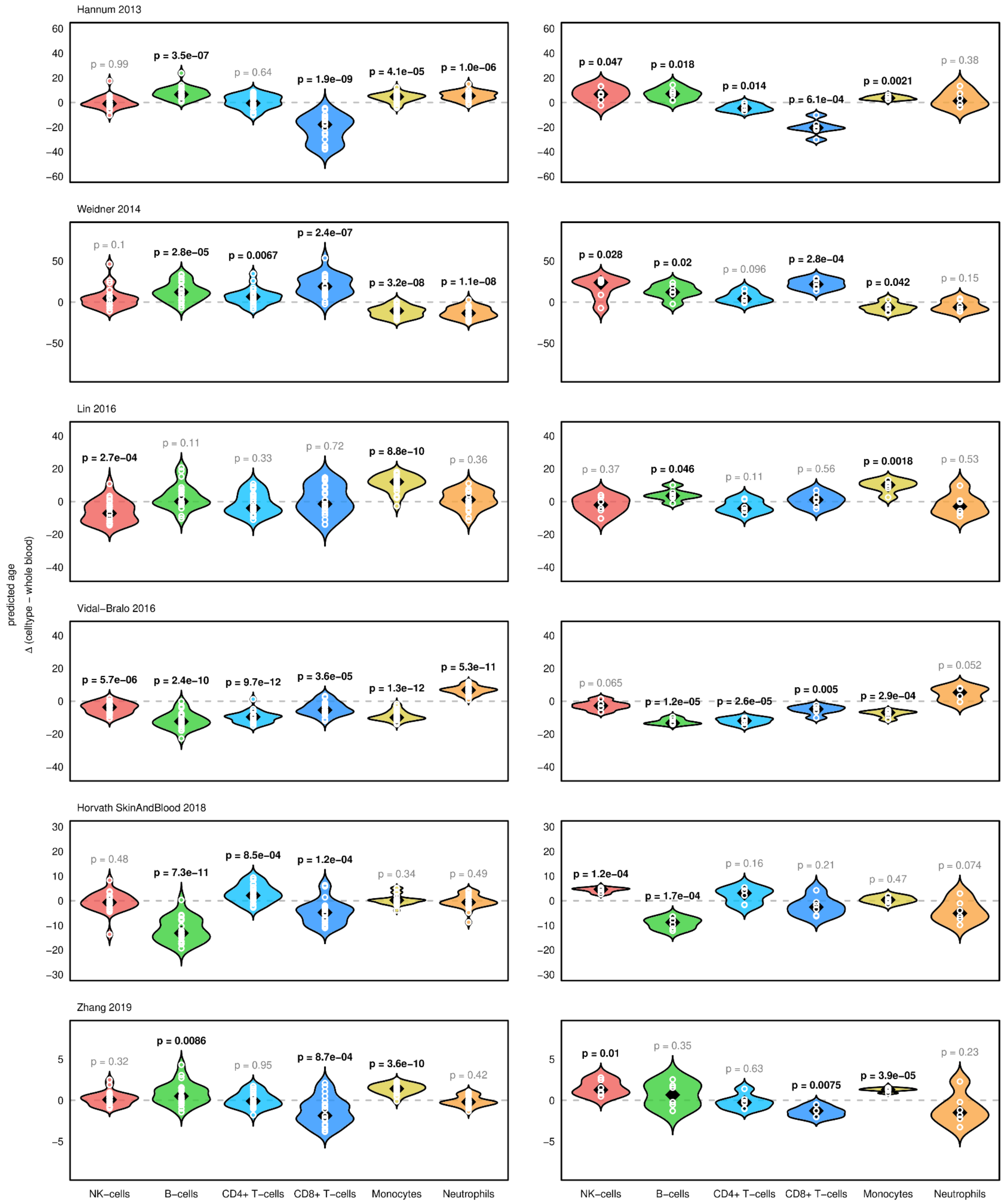

Figure S5 Predictions made by chronological epigenetic clocks for different WBC subtypes. Violin plots of epigenetic age differences between WBC subtypes and matched whole blood samples in Wang et al. (Wang et al. 2023) (left) and Reinius et al. (Reinius et al. 2012) (right) datasets. Numbers above violin plots depict p-values (bold - $p < 0.05$) of Student's paired t-test between each WBC subtype compared to whole blood, matched by donor. x-axis: WBC subtype; y-axis: epigenetic age deviation of WBC subtype from whole blood.

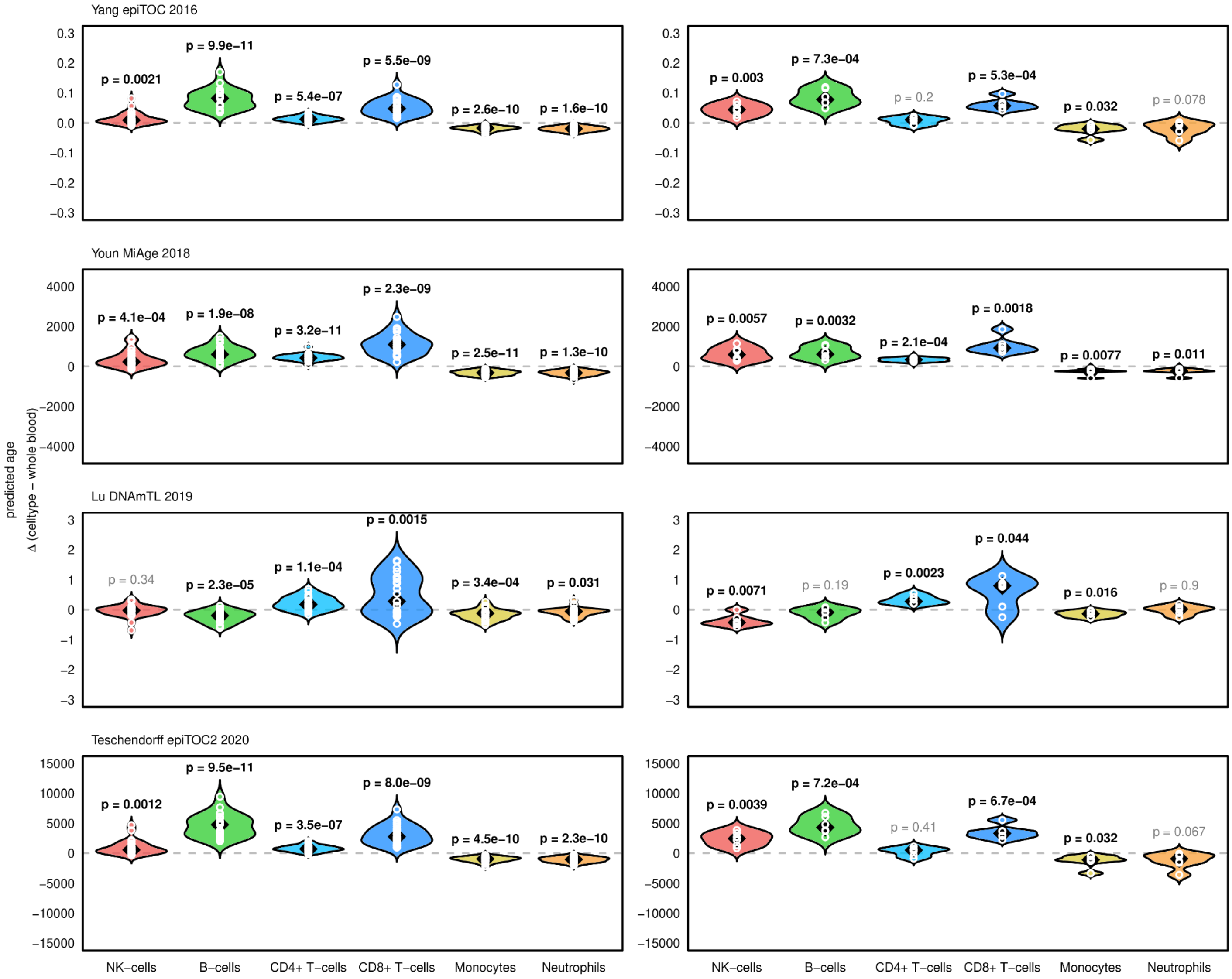

Figure S6 Predictions made by mitotic epigenetic clocks for different WBC subtypes. Violin plots of epigenetic age differences between WBC subtypes and matched whole blood samples in Wang et al. (Wang et al. 2023) (left) and Reinius et al. (Reinius et al. 2012) (right) datasets. Numbers above violin plots depict p-values (bold - $p < 0.05$) of Student’s paired t-test between each WBC subtype compared to whole blood, matched by donor. x-axis: WBC subtype; y-axis: epigenetic age deviation of WBC subtype from whole blood.

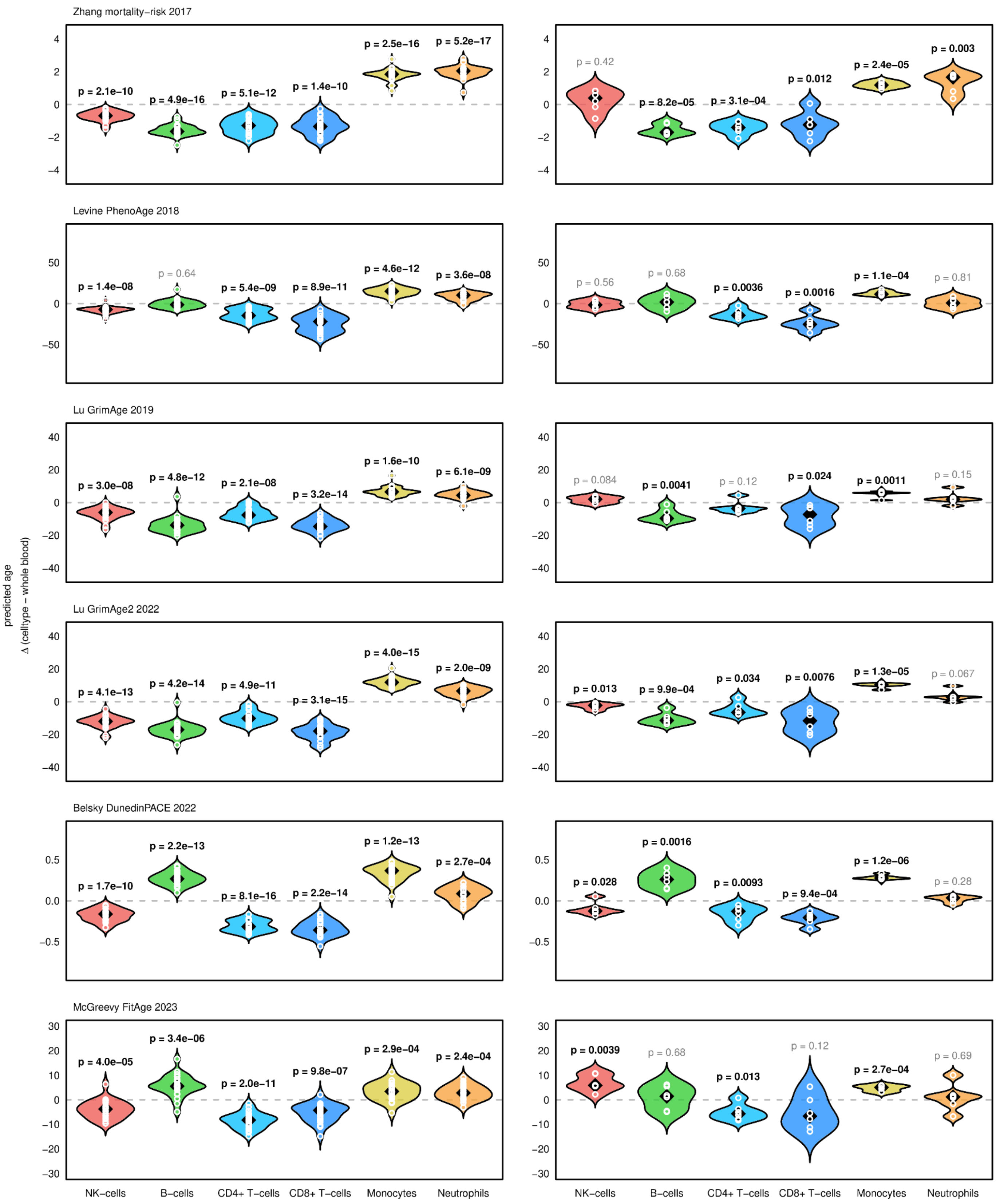

Figure S7 Predictions of biological and mortality epigenetic clocks across different WBC subtypes. Violin plots of epigenetic age differences between WBC subtypes and matched whole blood samples in Wang et al. (Wang et al. 2023) (left) and Reinius et al. (Reinius et al. 2012) (right) datasets. Numbers above violin plots depict p-values (bold - $p < 0.05$) of Student's paired t-test between each WBC subtype compared to whole blood, matched by donor. x-axis: WBC subtype; y-axis: epigenetic age deviation of WBC subtype from whole blood.

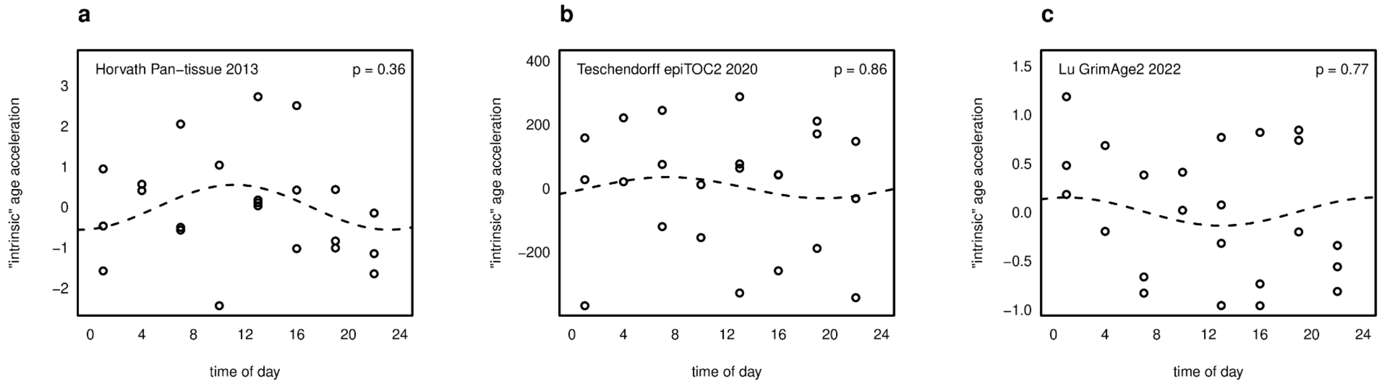

d

	Hannum 2013	Horvath pan−tissue 2013	Weidner 2014	Lin 2016	Vidal−Bralo 2016	Horvath SkinAndBlood 2018	Zhang 2019
p−value	0.79	0.36	0.85	0.29	0.57	0.92	0.67
R^2	0.02	0.1	0.02	0.12	0.05	0.0088	0.04
mesor	−0.01	−0.0056	0.03	0.02	−0.0072	−0.0028	0.0015
amplitude	0.21	0.55	0.45	0.94	0.3	0.09	0.04
acrophase	19:43	11:10	5:23	9:31	21:24	21:01	0:29

Chronological clocks

	Yang epiTOC 2016	Youn MiAge 2018	Lu DNAmTL 2019	Teschendorff epiTOC2 2020
p−value	0.87	0.88	0.19	0.86
R^2	0.01	0.01	0.15	0.01
mesor	3.2e−05	0.5	0.0011	1.87
amplitude	0.00056	7.27	0.02	33.3
acrophase	7:18	6:17	7:23	7:26

Mitotic clocks

	Zhang mortality−risk 2017	Levine PhenoAge 2018	Lu GrimAge 2019	Lu GrimAge2 2022	Belsky DunedinPACE 2022	McGreevy FitAge 2023
p−value	0.8	0.5	0.82	0.77	0.77	0.78
R^2	0.02	0.07	0.02	0.03	0.03	0.02
mesor	−0.00042	−0.03	0.0034	0.0063	−0.00022	0.005
amplitude	0.01	0.58	0.12	0.14	0.0036	0.14
acrophase	20:31	20:01	0:07	0:60	19:05	0:32

Biological clocks

Figure S8 Epigenetic age oscillations after adjustment for WBC subtype proportions. (a-c) 24 hr dynamics of epigenetic age predictions in WBC-Neu dataset regressed on chronological age and cell type proportions (see methods) for Horvath pan-tissue 2013 (a), Teschendorff epiTOC2 2020 (b), and Lu GrimAge2 2022 (c) clocks. Lines depict cosinor regression fits with solid lines indicating oscillation significance ($p < 0.05$). x-axis: time of day; y-axis: epigenetic age. (d) Estimated oscillation parameters of the 17 tested epigenetic clocks after adjustment for WBC subtypes.

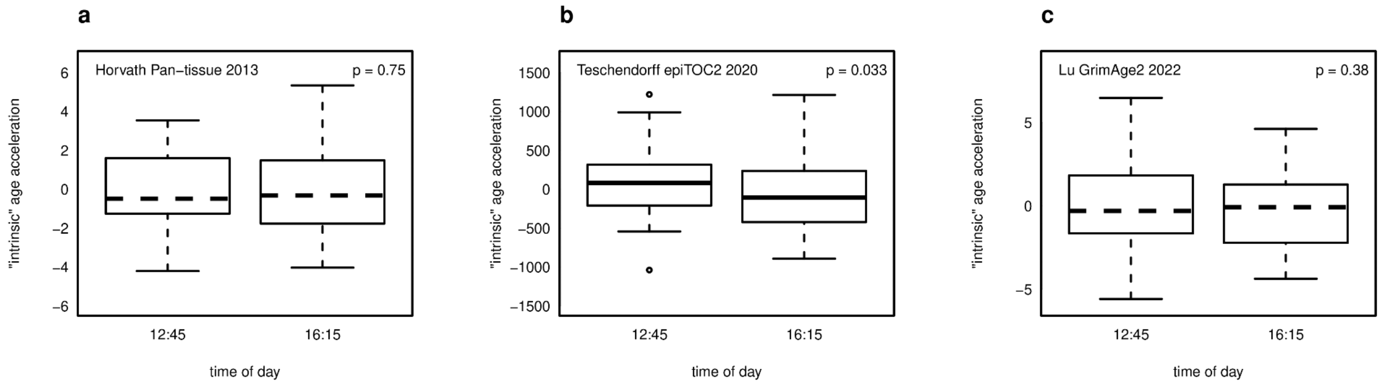

d

	Hannum 2013	Horvath pan-tissue 2013	Weidner 2014	Lin 2016	Vidal–Bralo 2016	Horvath SkinAndBlood 2018	Zhang 2019	Yang epiTOC 2016	Youn MiAge 2018	Lu DNAmTL 2019	Teschendorff epiTOC2 2020	Zhang mortality–risk 2017	Levine PhenoAge 2018	Lu GrimAge 2019	Lu GrimAge2 2022	Belsky DunedinPACE 2022	McGreevy FitAge 2023
p−value	**0.026**	0.75	0.98	0.62	0.4	0.13	0.95	**0.025**	**0.0035**	0.16	**0.033**	0.66	0.12	0.52	0.38	0.14	0.22
Δ 16:15–12:45	**−0.87**	0.15	0.04	−0.39	−0.4	−0.53	0.0085	**−0.0024**	**−31.3**	0.04	**−126**	0.01	1.19	−0.31	−0.4	0.01	−0.52
	Chronological clocks							Mitotic clocks				Biological clocks					

Figure S9 Time-of-day effects after adjustment for WBC subtype proportions. (a-c) Boxplots of epigenetic age predictions regressed on chronological age and cell type proportions at two time points - 12:45 and 16:15 (Apsley et al. 2023) for Horvath pan-tissue 2013 (a), Teschendorff epiTOC2 2020 (b), and Lu GrimAge2 2022 (c) clocks. Solid median lines indicate statistically significant differences ($p < 0.05$, paired Student's t-test). x-axis: time of day; y-axis: epigenetic age. (d) Paired Student's t-test p-values and pairwise mean differences of adjusted epigenetic age between 12:45 and 16:15, matched by donor. Bold values indicate significant oscillations ($p < 0.05$).

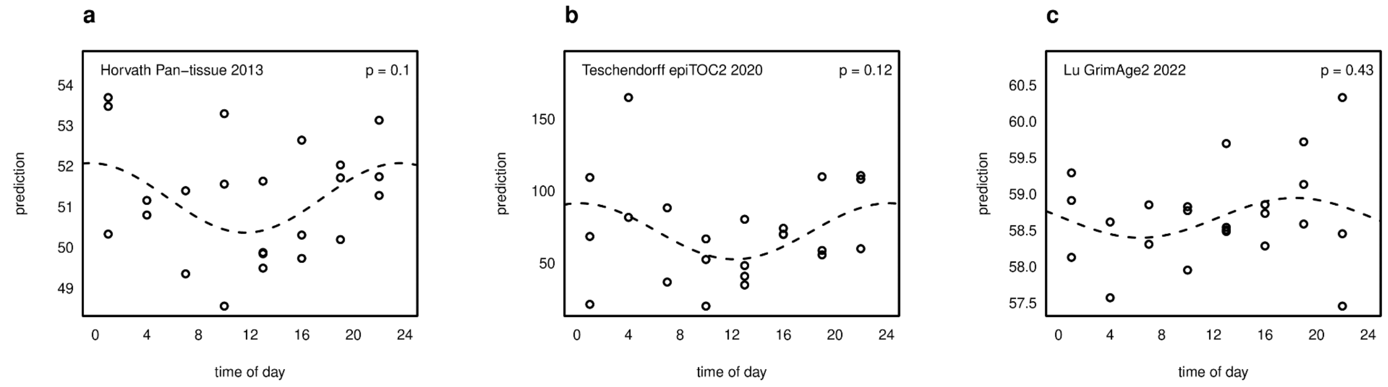

d

	Hannum 2013	Horvath pan-tissue 2013	Weidner 2014	Lin 2016	Vidal-Bralo 2016	Horvath SkinAndBlood 2018	Zhang 2019
p−value	0.88	0.1	0.42	0.099	0.18	0.9	0.99
R^2	0.01	0.2	0.08	0.21	0.16	0.01	0.0015
mesor	60.6	51.2	15.9	53.9	59.9	51.6	62.7
amplitude	0.15	0.86	0.62	0.89	0.42	0.08	0.01
acrophase	5:09	23:38	21:35	7:28	9:52	17:15	18:35

Chronological clocks

	Yang epiTOC 2016	Youn MiAge 2018	Lu DNAmTL 2019	Teschendorff epiTOC2 2020
p−value	0.18	0.068	0.79	0.12
R^2	0.16	0.24	0.02	0.19
mesor	0.03	450	7.05	71.9
amplitude	0.00034	5.68	0.0058	19.4
acrophase	0:08	4:07	5:52	0:12

Mitotic clocks

	Zhang mortality−risk 2017	Levine PhenoAge 2018	Lu GrimAge 2019	Lu GrimAge2 2022	Belsky DunedinPACE 2022	McGreevy FitAge 2023
p−value	**0.011**	0.83	0.4	0.43	0.74	0.4
R^2	**0.36**	0.02	0.09	0.08	0.03	0.09
mesor	**−1.08**	54	53.1	58.7	1.01	49.2
amplitude	**0.07**	0.21	0.28	0.27	0.0041	0.31
acrophase	**13:44**	0:30	17:12	18:20	6:42	16:49

Biological clocks

Figure S10 Epigenetic age oscillations in neutrophils of a 52 yr old individual. (a-c) Epigenetic age predictions using Horvath pan-tissue 2013 (a), Teschendorff epiTOC2 2020 (b), and Lu GrimAge2 2022 (c) clocks. Lines depict cosinor regression fits, solid lines indicate significant oscillations ($p < 0.05$). x-axis: time of day; y-axis: epigenetic age. (d) Estimated oscillation parameters of the 17 tested epigenetic clocks. Bold values indicate significant oscillations ($p < 0.05$).

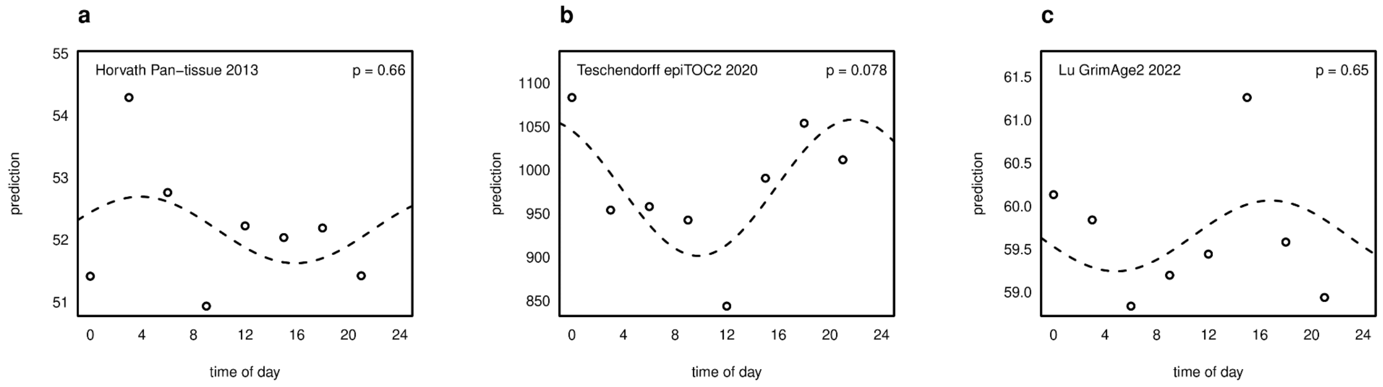

d

	Hannum 2013	Horvath pan-tissue 2013	Weidner 2014	Lin 2016	Vidal-Bralo 2016	Horvath SkinAndBlood 2018	Zhang 2019
p−value	0.43	0.66	0.12	0.4	0.59	0.11	0.68
R^2	0.29	0.15	0.57	0.31	0.19	0.58	0.14
mesor	46.2	52.2	30.3	49.3	61.4	52.2	61.5
amplitude	0.28	0.53	2.25	0.91	0.29	0.55	0.08
acrophase	7:25	3:53	3:33	5:07	6:57	16:06	19:41

Chronological clocks

	Yang epiTOC 2016	Youn MiAge 2018	Lu DNAmTL 2019	Teschendorff epiTOC2 2020
p−value	**0.025**	0.29	0.86	0.078
R^2	**0.77**	0.39	0.06	0.64
mesor	**0.05**	565	6.92	980
amplitude	**0.0012**	6.39	0.0069	78.3
acrophase	**21:25**	2:36	2:29	21:48

Mitotic clocks

	Zhang mortality−risk 2017	Levine PhenoAge 2018	Lu GrimAge 2019	Lu GrimAge2 2022	Belsky DunedinPACE 2022	McGreevy FitAge 2023
p−value	0.67	0.87	0.47	0.65	0.24	0.44
R^2	0.15	0.06	0.26	0.16	0.44	0.28
mesor	−0.04	42.8	55.6	59.6	0.8	56.5
amplitude	0.01	0.32	0.5	0.41	0.02	0.52
acrophase	8:13	17:23	18:52	16:50	23:31	0:53

Biological clocks

Figure S11 Epigenetic age oscillations in neutrophils of a 54 yr old individual. (a-c) Epigenetic age predictions using Horvath pan-tissue 2013 (a), Teschendorff epiTOC2 2020 (b), and Lu GrimAge2 2022 (c) clocks. Lines depict cosinor regression fits, solid lines indicate significant oscillations ($p < 0.05$). x-axis: time of day; y-axis: epigenetic age. (d) Estimated oscillation parameters of the 17 tested epigenetic clocks. Bold values indicate significant oscillations ($p < 0.05$).

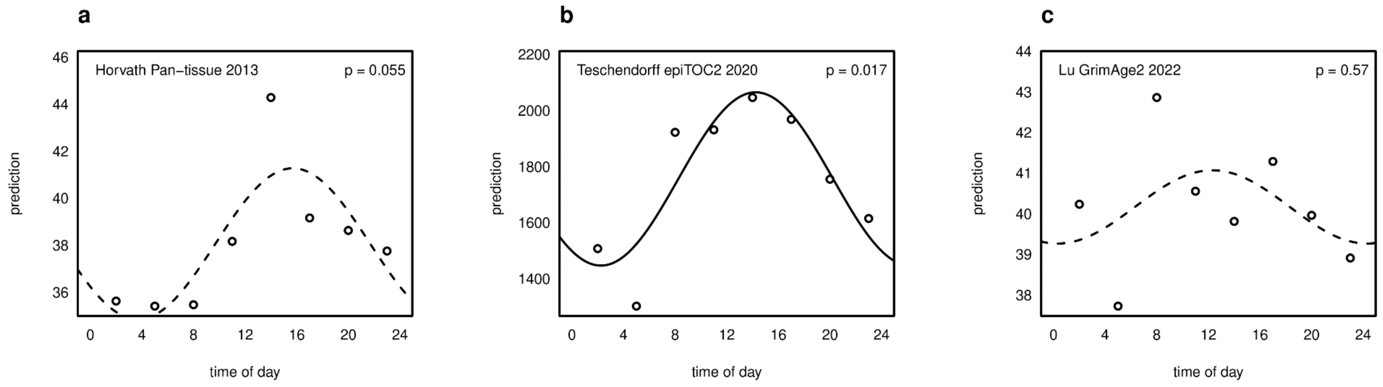

d

Chronological clocks

	Hannum 2013	Horvath pan-tissue 2013	Weidner 2014	Lin 2016	Vidal-Bralo 2016	Horvath SkinAndBlood 2018	Zhang 2019
p−value	0.78	0.055	0.19	0.38	0.11	0.68	0.99
R^2	0.1	0.69	0.49	0.32	0.59	0.14	0.0044
mesor	26.9	38.1	9.17	14.3	47.5	21.6	51.1
amplitude	0.59	3.21	0.97	2.19	1.19	0.62	0.02
acrophase	12:59	15:42	10:33	14:59	10:55	9:28	13:00

Mitotic clocks

	Yang epiTOC 2016	Youn MiAge 2018	Lu DNAmTL 2019	Teschendorff epiTOC2 2020
p−value	**0.039**	0.21	0.42	**0.017**
R^2	**0.73**	0.46	0.3	**0.8**
mesor	**0.07**	419	7.71	**1756**
amplitude	**0.0058**	13.7	0.05	**309**
acrophase	**14:16**	14:33	15:49	**14:15**

Biological clocks

	Zhang mortality−risk 2017	Levine PhenoAge 2018	Lu GrimAge 2019	Lu GrimAge2 2022	Belsky DunedinPACE 2022	McGreevy FitAge 2023
p−value	0.94	0.79	0.32	0.57	**0.0062**	0.6
R^2	0.03	0.09	0.36	0.2	**0.87**	0.18
mesor	0.38	35.6	38	40.2	**0.92**	33.2
amplitude	0.01	0.74	1.71	0.9	**0.03**	0.78
acrophase	0:50	13:21	13:05	12:19	**8:43**	15:44

Figure S12 Epigenetic age oscillations in neutrophils of a 30 yr old individual. (a-c) Epigenetic age predictions using Horvath pan-tissue 2013 (a), Teschendorff epiTOC2 2020 (b), and Lu GrimAge2 2022 (c) clocks. Lines depict cosinor regression fits, solid lines indicate significant oscillations ($p < 0.05$). x-axis: time of day; y-axis: epigenetic age. (d) Estimated oscillation parameters of the 17 tested epigenetic clocks. Bold values indicate significant oscillations ($p < 0.05$).
