# Supplementary material for: Epigenetic age oscillates during the day
Source: Aging Cell. 2024 Apr 18;23(7):e14170. doi: 10.1111/acel.14170 (PMC11258449; doi:10.1111/acel.14170)
Supplement: Supplementary file 3 — Data S1. [file ACEL-23-e14170-s002.pdf]

Supporting Information

Experimental Methods

Sample collection, bisulfite conversion, and microarray experiments

Twenty peripheral blood samples were collected every 3 hrs for 72 hrs from a 52-year-old Caucasian male. Collection started at circadian time 13 (CT13), corresponding to 1PM local time with one missing time point at CT52. At each time point eight milliliters of blood were collected in EDTA Vacutainers™. Neutrophils were isolated from the whole blood by magnetically activated cell sorting with an EasySep™ Direct Human Neutrophil Isolation Kit (STEMCELL Technologies, BC, Canada). After negative selection for neutrophils, both the purified neutrophils and the follow through of WBC-Neu fraction were washed with phosphate-buffered saline, pelleted, and snap-frozen in liquid nitrogen. Ten milliliters of blood from a healthy 54-year old Caucasian male were collected every 3 hrs for 24 hrs in EDTA Vacutainers™ and processed as described above using magnetically activated cell sorting protocol. Ten milliliters of blood were also collected from a healthy 30-year old Caucasian male every 3 hrs for 24 hrs in heparin Vacutainers™. Immediately after collecting, Optilyse C™ (Beckman Coulter, CA, USA), was added to each of these samples according to manufacturer's recommendation both for erythrolysis and mild fixation of the WBCs. These samples were then subsequently sorted for neutrophils via fluorescence activated cell sorting using CD15-APC, CD16-PE, and CD45- PE-Cy™7 (BD Pharmingen™, NJ, USA). All samples were snap-frozen and stored at −80 °C. DNA extraction was performed with NucleoSpin® Blood XL and Tissue kit (Macherey-Nagel, Westfalen, Germany) prior to downstream experiments.

For each time point 750 ng of genomic DNA were bisulfite-converted using an EZ DNA Methylation™ Kit (Zymo) according to the manufacturer's protocol for the HumanMethylation450k BeadChip (samples from 52 yr old donor) and HumanMethylationEPIC BeadChip (54 and 30 yr old donors) (Illumina, CA, USA), with the modifications suggested by the manufacturer for a higher conversion efficiency. 7.5 μL of M-dilution buffer was used for the reaction, which was incubated at 42 °C for 30 min prior to addition of the CT-Conversion Reagent. A total of 185 μL of the M-dilution buffer was used in the preparation of the CT-Conversion Reagent, and 97.5 μL of the reagent was added per reaction. All HumanMethylation arrays were performed in technical duplicates or triplicates using 500 ng of the bisulfite-converted genomic DNA at The Centre for Applied Genomics (Toronto).

Data preparation and quality control

All Illumina HumanMethylation microarray datasets were processed using the "minfi" package (Aryee et al. 2014). First, all samples having more than 3% of probes with poor detection p-values (detection $p < 0.05$) (Heiss & Just 2019) were removed as outliers. Then array normalization was performed using "noob" background correction (Triche et al. 2013) followed by functional normalization (Fortin et al. 2014), and signals from the methylated and unmethylated channels were combined to obtain the methylation beta values. Additional outliers were identified by measuring Pearson's correlations among all the sample pairs and removing samples with an average inter-sample correlation value more than three standard deviations below the mean. Finally, for the datasets that contained technical replicates for every sample, samples with only a single replicate remaining after quality control were discarded.

Estimation of cell type proportions

Cell type proportions were estimated using Houseman's method (Houseman et al. 2012). All analyses used Reinius et al. dataset (Reinius et al. 2012) as a reference, except the estimation of neutrophil proportion in WBC-Neu dataset, where an "enhanced cell deconvolution" reference by Salas et al.

(Salas et al. 2022) was used instead. Cell type proportion estimates were obtained for each technical replicate separately and averaged using the mean.

Epigenetic clock measurements

Epigenetic age estimates for Hannum 2013 (Hannum et al. 2013), Horvath pan-tissue 2013 (Horvath 2013), Horvath SkinAndBlood 2018 (Horvath 2013), Zhang 2019 (Zhang et al. 2019), Yang epiTOC 2016 (Yang et al. 2016), Lu DNAmTL 2019 (Lu, Seeboth, et al. 2019) Teschendorff epiTOC2 2020 (Teschendorff 2020), Levine PhenoAge 2018 (Levine et al. 2018), Lu GrimAge 2019 (Lu, Quach, et al. 2019), Lu GrimAge2 2022 (Lu et al. 2022), and McGreevy FitAge 2023 (McGreevy et al. 2023) clocks were obtained by uploading the raw datasets to an online DNA Methylation Age Calculator developed by the Clock Foundation (https://dnamage.clockfoundation.org/), and following their instruction manuals. Youn MiAge 2018 (Youn & Wang 2018) clock estimates were calculated according to the scripts provided by the authors (http://www.columbia.edu/~sw2206/softwares.htm). Belsky DunedinPACE 2022 clock (Belsky et al. 2022) estimates were obtained via the R package "DunedinPACE" (https://github.com/danbelsky/DunedinPACE). Epigenetic age predictions for the remaining clocks (Weidner 2014 (Weidner et al. 2014), Lin 2016 (Lin et al. 2016), Vidal-Bralo 2016 (Vidal-Bralo et al. 2016) and Zhang mortality-risk 2017 (Zhang et al. 2017)) were obtained via the R package "methylCIPHER" (Thrush et al. 2022) (https://github.com/MorganLevineLab/methylCIPHER). Epigenetic age estimates for the PC versions of the clocks (Higgins-Chen et al. 2022) were calculated according to the instructions provided by the authors (https://github.com/MorganLevineLab/PC-Clocks).

Cell type adjustment of epigenetic age for all the clocks was performed by following the strategy outlined in a study by Chen et.al (Chen et al. 2016). Briefly, epigenetic age acceleration after adjustment is defined as residuals remaining after the regression of the predicted age on real chronological age and the predicted proportions of CD4+ T-lymphocytes, CD8+ naive T-lymphocytes, CD8+ exhausted T-lymphocytes, NK-cells, plasma blasts, monocytes, and granulocytes, as returned by the Clock Foundation online calculator.

Epigenetic age estimates were obtained for each technical replicate separately and were then averaged using the mean.

Detection of 24 hr oscillation patterns

A cosinor model (Cornelissen 2014) with a fixed 24 hr period was used in order to identify circadian oscillations in the variable of interest (either a methylation level of individual cytosines, cell type proportion estimates or epigenetic age measurements). First, for all samples, the measurements across all technical replicates were averaged and the mean used for further analysis. The phase, mesor, and amplitude were modeled as a linear combination of sine and cosine terms:

$$y = b_0 + b_1 sin\,(2\pi \cdot \frac{CT}{24}) + b_2 cos\,(2\pi \cdot \frac{CT}{24}) + \epsilon$$

where *y* is the estimated measure of interest, b_i are regression coefficients, *CT* is the circadian time, and *ε* is the error term. The p-values were obtained by comparing this model to the null intercept-only model using an F test.

Circadian 24 hr period oscillations in a combined analysis of purified neutrophil datasets allowed the model to estimate separate MESOR, amplitude, and acrophase values for each of the three individuals, by adding a covariate for the donor as well as interaction terms between the donor and cosinor sine and cosine terms. For this analysis the p-values were obtained by comparing the cosinor model with added interaction terms to the null model with a single covariate for the donor effect, using an F test.

Epigenetic age of WBC subtypes

The comparison of epigenetic age between purified cell subtypes and whole blood was done using Wang et al. (Wang et al. 2023) and Reinius et al. (Reinius et al. 2012) DNA modification datasets. Whole blood samples in the Wang et al. dataset were composed of both HumanMethylation450k as well as HumanMethylationEPIC arrays and, in order to avoid technical biases between purified WBC subtypes and whole blood, all the EPIC arrays were discarded. In addition, only the donors that, after quality control, had remaining DNA modification array measurements across all 6 investigated WBC subtypes and whole blood were selected for analysis. This resulted in 23 and 6 remaining donors for the Wang et al. and Reinius et al. datasets, respectively. The cell type specific epigenetic age deviation (acceleration or deceleration, compared with whole blood) was obtained by taking the means of donor-matched epigenetic age differences between each WBC subtype and whole blood. The p-values were obtained using a paired Student's t-test.

The numbers reported in the main text reflect the average differences across both investigated datasets.

Epigenetic age time of day effects

Epigenetic clock oscillations in a populational setting were investigated using Apsley et al. dataset (Apsley et al. 2023). The original study investigated the effects of acute stress and contained DNA modification measurements for 34 stress and 14 control sessions, in all of which DNA modifications were monitored across 4 different time points (at 11:30, 12:45, 13:45, and 16:15). For the purposes of the present study the epigenetic age estimates were compared between two time points - 12:45 (time closest to the observed epigenetic age oscillation peak) and 16:15. After quality control, only the sessions with available DNA modification array measurements at both of the specified time points were selected. This resulted in 21 stress and 11 control sessions. The epigenetic clock deviations between 12:45 and 16:15 were estimated by taking the mean of session-matched epigenetic age differences. The p-values were obtained using a paired Student's t-test. Due to the small sample size of the control group the analysis did not take the stress variable into account.

Data availability

Raw Illumina HumanMethylation array files generated by this study are available via the Gene Expression Omnibus (GEO) under accession numbers GSE247197 (WBC-Neu, 52 yr old), GSE247195 (neutrophils, 54 yr old), and GSE247193 (neutrophils, 30 yr old). Neutrophil dataset for the 52 yr old participant was published previously (Oh et al. 2019) and can be obtained through GEO via accession number GSE83944. The raw data files for all the re-analyzed publicly available datasets are also accessible through GEO: GSE227809 (PBMC at 4 time points, Apsley et al. (Apsley et al. 2023)), GSE35069 (WBC subtypes, Reinius et al. (Reinius et al. 2012)), GSE224807 (WBC subtypes, Wang et al. (Wang et al. 2023)), and GSE87571 (Whole blood, Johansson et al. (Johansson et al. 2013)).

Code availability

R scripts for producing the results and images are deposited on GitHub at https://github.com/karoliskoncevicius/pub_epiclock_oscs.
